# Human papillomavirus (HPV) infection and intraepithelial neoplasia and invasive cancer of the uterine cervix: a case-control study in Zaragoza, Spain

**DOI:** 10.1186/1750-9378-3-8

**Published:** 2008-05-29

**Authors:** Milagros Bernal, Isabel Burillo, Jose I Mayordomo, Manuel Moros, Rafael Benito, Joaquina Gil

**Affiliations:** 1 Department of Microbiology, Preventive Medicine and Public Health, University of Zaragoza School of Medicine, Zaragoza, Spain; 2 Division of Medical Oncology, University Hospital, Zaragoza, Spain; 3 Division of Surgical Pathology, University Hospital, Zaragoza, Spain; 4 Servicio de Oncología Médica, Hospital Clínico Universitario, Av. San Juan Bosco, 15, 50009, Zaragoza, Spain

## Abstract

**Introduction:**

The raw incidence of cancer of the uterine cervix is Spain is 7,8 per 100.000 inhabitants (adjusted incidence is 5.6). The incidence of this tumor is still low, but a steady increase has been seen, probably related to increasing risk factors.

**Aim:**

To determine the frequency of infection by different types of human papillomavirus (HPV) in Papanicolau smears from women with and without cancer of the uterine cervix in Spain.

**Patients and methods:**

A case-control study was performed in women with and without cervical cancer from Zaragoza, Spain. Pap smears from 600 cases (540 women with cervical intraepithelial neoplasms (CIN) and 60 with invasive cancer) and 1200 controls (women without those lesions) were tested by polymerase chain reaction (PCR) and typed by oligonucleotide microarray-based detection.

**Results:**

HPV was detected in 93.3% of all samples with invasive cancer versus 17.5% of controls. OR for invasive cancer was 55 (95% CI 21.5–140,5). Statistically significant associations were also found for different grades of cervical dysplasia.

**Conclusion:**

The strong association found between HPV infection, specifically types 16 and 18 and cancer of the uterine cervix in Zaragoza, Spain, stresses the importance of ongoing efforts to institute a vaccine program with recently approved HPV vaccines in order to prevent cervical cancer in this population.

## Background

Cancer of the uterine cervix is more frequent in developing countries than in the Western world. Spain, a country where screening through Papanicolau smears has been performed for more than 30 years now, has a low raw incidence of 7.8 in 2000 (age-adjusted rate is 5.6) [1].

In the last decade, an increasing incidence of cervical cancer has been noted, most likely related to increasing numbers of young women with risk factors, namely sexual promiscuity and prostitution.

The fact that these high-risk women, some of whom are foreign immigrants, are not adhering to screening programs, stresses the importance of developing alternative strategies for prevention.

While there are many genotypes of human papillomavirus and most of them are frequently found in Pap smears of healthy women, two genotypes, 16 and 18, of human papillomavirus have been consistently associated to intraepithelial neoplasia and invasive cancer of the uterine cervix in Western populations [2] and have been included in papillomavirus vaccines. However, it must be noted that additional genotypes that are not included in the first generation of human papillomavirus vaccines, that has just been approved for clinical use, are frequently associated to intraepithelial and invasive cervical neoplasia in South America and Asia.

We have performed a case-control study to assess the incidence of infection by different HPV types in women with and without cervical dysplasia and carcinoma. Following PCR-based detection of HPV, oligonucleotide microarray-based detection was used for genotyping, given its reliability and ease of use.

## Methods

Following approval by the Institutional review Board of the University of Zaragoza, the study included six hundred consecutive women with some degree of cervical dysplasia or neoplasia (CIN I, CIN II, CIN III and invasive cancer) excluding atypical squamous cells of uncertain significance (ASCUS) who had Pap smears at the University Hospital of Zaragoza, Spain from 1999 to 2005. Cytological diagnoses were required to have been subsequently confirmed by histology for study entry.

Controls were 1200 age-matched women who had no cervical dysplasia, neoplasia or ASCUS in the Pap smear, for a case:control ratio of 1:2.

In addition to standard cytologic examination, all Pap smears from cases and controls underwent DNA extraction and were tested for HPV by polymerase chain reaction (PCR) and typed by oligonucleotide microarray-based detection [3] with positive and negative controls.

Statistical analysis included a logistic regression analysis adjusted for age, calculating odds ratio (OR) for each type of cervical lesion (CIN I, CIN II; CIN III and invasive cancer) and 95% confidence intervals in women with and without HPV positivity in the Pap smear.

## Results

The study population included 1800 women. Median age was 36 years. There were 3.3% foreign-born individuals. The number of samples in each diagnostic group was: 120 patients (20%) with CIN I, 210 (35%) with CIN II, 210 (35%) with CIN III and 60 (10%) with invasive cervical carcinoma. All these cytological diagnoses had been subsequently confirmed by histology. The frequency of HPV positivity according to type of cervical lesion is shown in Table 1. Overall, 79% of cases and 17% of controls tested positive for HPV. According to the type of lesion, 66% of patients with CIN I tested positive for HPV, as did 71% of those with CIN II, 90% of those with CIN III and 90% of patients with invasive cervical carcinoma.

**Table 1:** Frequency of HPV positivity according to type of cervical lesion found in Pap smears.

Pathological diagnosis	Number of patients	Number (%) HPV +
CIN I	120	80 (66%)
CIN II	210	150 (71%)
CIN III	210	190 (90%)
Invasive carcinoma	60	56 (93%)
Total cases	600	476 (79%)
Controls	1200	210 (17%)

As for HPV types (Table 2), the most frequent type of HPV found in women with CIN I, CIN II, CIN III and invasive carcinoma was HPV-16, followed by HPV-18. In patients with CIN, these two genotypes comprised 55% of HPV infections detected, versus 80% in patients with CIN II and 90% in patients with CIN III. In patients with invasive carcinoma, HPV-16 and HPV-18 comprised 98% of all HPV infections detected.

**Table 2:** Most frequent HPV genotypes found in the study according to the type of cervical lesion.

	Pathological diagnosis				
Genotype found	CIN I	CIN II	CIN III	Invasive carcinoma	No cancer (control)

HPV-16	30%	30%	80%	90%	1%
HPV-18	25%	50%	10%	8%	0
HPV-1	10%	5%	0%	0%	30%
HPV-4	0%	3%	2%	0%	12%
HPV-31	7%	6%	3%	0%	49%
HPV-54	27%	4%	2%	0%	12%
Others	1%	2%	3%	2%	26%

It is remarkable that HPV 16 and HPV 18 were infrequent among controls, comprising only 1% of all HPV infections.

Table 3 shows the odds ratio for each type of cervical lesion in women with or without HPV positivity. The odds ratio was higher for high-grade lesions, ranging from 6 for CIN I to 55 for infiltrating carcinoma.

**Table 3:** Odds ratio for each type of cervical lesion according to HPV status.

Type of lesion	Odds ratio	95% Confidence interval
CIN I	6.0	3.7–9.7
CIN II	10.6	7.2–15.6
CIN III	16.7	10.5–26.4
Invasive carcinoma	55.0	21.5–140.5

## Discussion

In the present study, the correlation between HPV and cervical neoplasia has been different for early lesions (odds ratio for CIN I = 6; odds ratio for CIN II = 10) than for higher-risk lesions (odds ratio for CIN III = 16; odds ratio for invasive carcinoma = 55).

Overall, the odds ratio found in our study is well within values reported in prior articles, ranging from a lowest value of 2 (95% confidence interval = 1.5–2.6) in a series of women with cancer of the uterine cervix from the United Kingdom to a high odds ratio of 813 (95% confidence interval = 168–3229) seen in another study [4]. Other authors found intermediate values, such as 18.5 (95% confidence interval = 5.9–57.6) [5] and 67.2 (95% confidence interval = 28.6–157.5) [6]. The odds ratio in women with HPV and coexisting HIV infection was found to be 16.8 (95% confidence interval = 7.0–40.3) [7].

Such large differences in studies that are otherwise concordant in showing a significant association between HPV infection and cervical cancer may well be due to differences in the type of pathological lesion, the viral load and the duration of infection.

In a study from Mexico [8], the odds ratio for CIN I in women with low viral load was 16.8 (95% confidence interval = 7.2–39), while the odds ratio for CIN II and CIN III in women with high viral load was 365 (95% confidence interval = 94.7–1412).

In the present study, a higher odds ratio was found for more severe cervical lesions, reaching a maximum of 55 for invasive carcinoma, a figure that is remarkably similar to that found in prior studies.

The fact that HPV detection was performed just after cytological examination, without the long storing periods seen in some prior studies [9] supports the reliability of the present data.

Both HPV-16 and HPV-18 were significantly more frequent in cases than in controls, as seen in prior reports [10-15]. There is a trend to increasing percentage of HPV-16 and HPV-18 infections in patients with CIN III (90%) as compared to those with CIN I (55%).

An interesting finding in our study is the high prevalence of HPV-54 in controls (12%) and patients with CIN I (27%). The fact that the prevalence of HPV-54 in patients with CIN III and invasive carcinoma was much lower (0% and 2%) suggests that this type has a low potential to induce invasive cancer.

Prior studies have found other HPV genotypes different from HPV-16 and HPV-18 to be related to cervical cancer in specific geographic areas [16]. That is the case for HPV-58, found frequently in women with cervical cancer in Central and Soutth America [17] and China. This suggests that the distribution of HPV types in women with squamous cell carcinoma is heterogeneous around the world.

As in other studies performed in Spain [18], HPV 16 and 18 are the only genotypes that are significantly associated to intraepithelial and invasive carcinoma of the uterine cervix. It is possible that significant immigration from South America and Asia might result in the appearance of additional oncogenic genotypes not included in current HPV vaccines.

It is remarkable that a significant percentage of healthy women (controls) harbour HPV (17% in the present study, similar to prior reports), even if the types were different from women with cervical lesions and the prevalence of types associated with cancer (namely HPV-16 and HPV-18) was very low.

The high frequency of HPV found in controls suggests that most were transient infections. Only women infected with high-risk HPV types and with ineffective immune response would develop neoplasia [19-21].

The distribution of HPV types associated to cervical cancer in each area of the world needs to be taken into account when designing HPV vaccines. This is an exciting time for prevention of cancer of the uterine cervix, when two HPV vaccines have already been proven to be effective in preventing cervical cancer, and may soon improve public health strategies against this disease, currently based solely on early detection by Pap smears.

## Conclusion

The strong association found in this report between HPV infection, specifically types 16 and 18 and cancer of the uterine cervix in Zaragoza, Spain, stresses the importance of ongoing efforts to institute a vaccine program with two recently approved HPV vaccines in order to prevent cervical cancer in this population.

## Authors' contributions

MB and IB designed the study, MM, RB and JG carried out the molecular genetic studies, JIM drafted the manuscript. All authors read and approved the final manuscript.
